# Plasticity of wheat seedling responses to K^+^ deficiency highlighted by integrated phenotyping of roots and root hairs over the whole root system

**DOI:** 10.1007/s44154-023-00083-4

**Published:** 2023-04-06

**Authors:** Ikram Madani, Jean-Benoît Peltier, Martin Boeglin, Hervé Sentenac, Anne-Aliénor Véry

**Affiliations:** Institut des Sciences des Plantes de Montpellier, UMR 5004 CNRS- 386 INRAE- Université Montpellier- Institut Agro, Campus SupAgro-INRAE Bat 7, Place Viala, Montpellier, 34060 Cedex 2 France

**Keywords:** K^+^ deficiency, Root hair, Root system Phenotyping, Wheat, Rhizobox, Root plasticity

## Abstract

The availability in the soil of potassium (K^+^), a poorly mobile macronutrient required in large quantities for plant growth, is generally suboptimal for crop production in the absence of fertilization, making improvement of the ability of crops to adapt to K^+^ deficiency stress a major issue. Increasing the uptake capacity of the root system is among the main strategies to achieve this goal. Here, we report an integrative approach to examine the effect of K^+^ deficiency on the development of young plant entire root system, including root hairs which are known to provide a significant contribution to the uptake of poorly mobile nutrients such as K^+^, in two genetically distant wheat varieties. A rhizobox-type methodology was developed to obtain highly-resolved images of root and root hairs, allowing to describe global root and root hair traits over the whole root system via image analysis procedures. The two wheat varieties responded differently to the K^+^ shortage: Escandia, a wheat ancestor, reduced shoot biomass in condition of K^+^ shortage and substantially increased the surface area of its root system, specifically by increasing the total root hair area. Oued Zenati, a landrace, conversely appeared unresponsive to the K^+^ shortage but was shown to constitutively express, independently of the external K^+^ availability, favorable traits to cope with reduced K^+^ availability, among which a high total root hair area. Thus, valuable information on root system adaptation to K^+^ deficiency was provided by global analyses including root hairs, which should also be relevant for other nutrient stresses.

**Supplementary Information:**

The online version contains supplementary material available at 10.1007/s44154-023-00083-4.

## Introduction

Together with leaf photosynthetic activity, the root system contributes to plant autotrophy by taking up nutrients and water from the soil. In most ecosystems, plant growth is notably limited by the low bio-availability in the soil of N, P and K. In conventional agriculture, intensive soil fertilization strongly increases crop yield, but this is achieved at high environmental costs, which are now widely considered as unsustainable. The evolution towards alternative models aiming at maintaining high yields while respecting the environment implies the implementation of science-based solutions. In this context, with the objective of contributing to a vital new green revolution, strong efforts have thus been aimed at characterizing the mechanisms underlying plant adaptation to low nutrient availability. They have actually provided evidence, in particular, that the genetic variation in root traits can be used to reduce fertilization inputs and increase crop yield in poor soils (Lynch 2011; White et al. 2013).

In the soil, the status, in terms of concentration and bioavailability, differ between nutrients (Marschner and Rengel 2012). K^+^ is poorly mobile due to interactions with soil minerals and organic matter (Jungk and Claassen 1997; Marschner and Rengel 2012). Its available concentration is most often in the 0.1–1 mM range. K^+^ acquisition by the root system requires its transfer to the root surface, which can occur both by mass flow (the movement of soil solution to the surface of the root system driven by the transpiratory water loss of the plant) and by diffusion along the concentration gradient created by the uptake by root periphery cells that depletes the soil solution within the rhizosphere at the root surface (White and Greenwood 2013). K^+^ deficiency in agriculture, although less analyzed than nitrogen and phosphorus deficiencies by plant nutrition biologists, is remarkably broad, affecting crops worldwide (Zörb et al. 2014; Ruan et al. 2015). Large agricultural areas of the world are indeed reported to be deficient in available K^+^, for instance two-thirds of the wheat belt of Southern Australia and three-fourth of the paddy soils in China (Mengel and Kirkby 2001; Römheld and Kirkby 2010; Zörb et al. 2014; Ruan et al. 2015; Thornburg et al. 2020). It is widely acknowledged that further research should be conducted on root responses to K^+^ deficiency to develop strategies to increase crop adaptation to this problem (Minjian et al. 2007; Zörb et al. 2014; Sustr et al. 2019; Thornburg et al. 2020).

Root traits influencing the acquisition of nutrient ions are very diverse and differ between plant species (White et al. 2013; Kellermeier et al. 2014). For example, efficient soil foraging, in a broad sense, is dependent on the root mass fraction (the proportion of total plant mass allocated to roots) as well as the root surface area, including root hairs. Efficient soil mining relies, for example, on expression of high affinity membrane transport systems in root periphery cells, as well as modification of rhizosphere physico-chemical conditions, via the secretion of organic acids, chelating molecules and enzymes, resulting in increased bioavailability of nutrient ions, and the exudation of compounds attracting and feeding beneficial microorganisms and symbionts (White et al. 2013). As a prerequisite of efficient nutrient uptake, the root area in contact with the soil has to be large to allow adequate quantities of the soil nutrients to reach the root surface. The roots of most plant species have the ability to produce root hairs which extend the absorptive surface of roots and may be regarded as a small-scale subsystem of roots foraging for nutrients (Jungk 2001). For poorly mobile nutrients like K^+^, root hairs are more efficient than the "naked" root cylinder/body in drawing advantage from the laws of diffusion due to their thinner diameter (Claassen and Steingrobe 1999; Jungk 2001). They express high-affinity nutrient uptake systems (Damiani et al. 2016; Bienert et al. 2021; Rongsawat et al. 2021) and have been assumed to have a higher influx per unit area than that of the root axis (Gahoonia and Nielsen 1998).

Consequences of K^+^ deficiency on root system architecture and root hair development have been little studied (Wang et al. 2016, 2021; Bienert et al. 2021). Reports, however, already exist in different species (Høgh‐Jensen & Pedersen 2003; Cao et al. 2013; Zhao et al. 2016; Sustr et al. 2019). An increase in the ratio of root to shoot biomass, indicative of a relative enhancement of the flux of photosynthates allocated to roots, when compared to shoots, and increases in root hair length and/or density have been mentioned (Høgh‐Jensen & Pedersen 2003; Hermans et al. 2006; Sustr et al. 2019). However, existing publications have not systematically reported such changes, revealing differences between species and even between genotypes within some species (Chen and Gabelman 2000; Desbrosses et al. 2003; Klinsawang et al. 2018; Sustr et al. 2019; Thornburg et al. 2020; Bienert et al. 2021). Information on root hair development responses in terms of changes in elongation and density is poor. Furthermore, apparently contradictory results can be found, which could result from differences in experimental approaches (e.g., with respect to the soil substrate, phenotyping procedure or applied K^+^ shortage concentration) or may reflect differences between plant species (Klinsawang et al. 2018; Bienert et al. 2021) or accessions: for example, either no change or an increase in root hair elongation was reported during K^+^ shortage in Arabidopsis (Desbrosses et al. 2003; Jung et al. 2009; Sustr et al. 2019). With respect to the response of root system development to K^+^ deficiency, investigations have mostly reported negative effects (Hermans et al. 2006; Thornburg et al. 2020). Altogether, the data reveal however that the responses to K^+^ deficiency are complex and can differ between root types, and also between species and accessions (Ashley et al. 2006; Jung et al. 2009; Kellermeier et al. 2013, 2014; Sustr et al. 2019; Thornburg et al. 2020). For instance, in *Arabidopsis thaliana* Col-0, K^+^ shortage conditions did not affect growth of the primary root, but decreased the elongation of first order lateral roots, and increased that—and the number of—the second order laterals (Kellermeier et al. 2014). But some other *A. thaliana* ecotypes (e.g. Catania-1) have been shown to display quite different responses to low K^+^, with arrest of primary root elongation in favor of lateral branching (Kellermeier et al. 2014). Taking stock of the current information in this domain, the conclusion is that the available knowledge on root responses to K^+^ deficiency is strongly lower in crop plants than in the model plant Arabidopsis and that, even in Arabidopsis, the characterization of this response remains fragmentary, with some results appearing contradictory (Sustr et al. 2019).

The aim of the present study was to investigate the consequences of K^+^ shortage on root and root hair development in a major crop, wheat. Our investigation of the consequences of K^+^ shortage on the root system aimed to provide a holistic view through an integrative analysis, by measuring the total absorptive surface area of the root system and the contribution of the root hair area to this total root system area. To our knowledge, this approach has not been used previously in the reports on root system responses to K^+^, N or P shortages. We developed a rhizobox-like methodology to achieve this goal and compared two subspecies of wheat (a landrace and a wheat ancestor) to test biological diversity in response to K^+^ deficiency in this crop.

## Results

### A rhizobox-type device for high-resolution phenotyping of root systems that allows integrated global analysis of root hairs

**Fig. 1:**
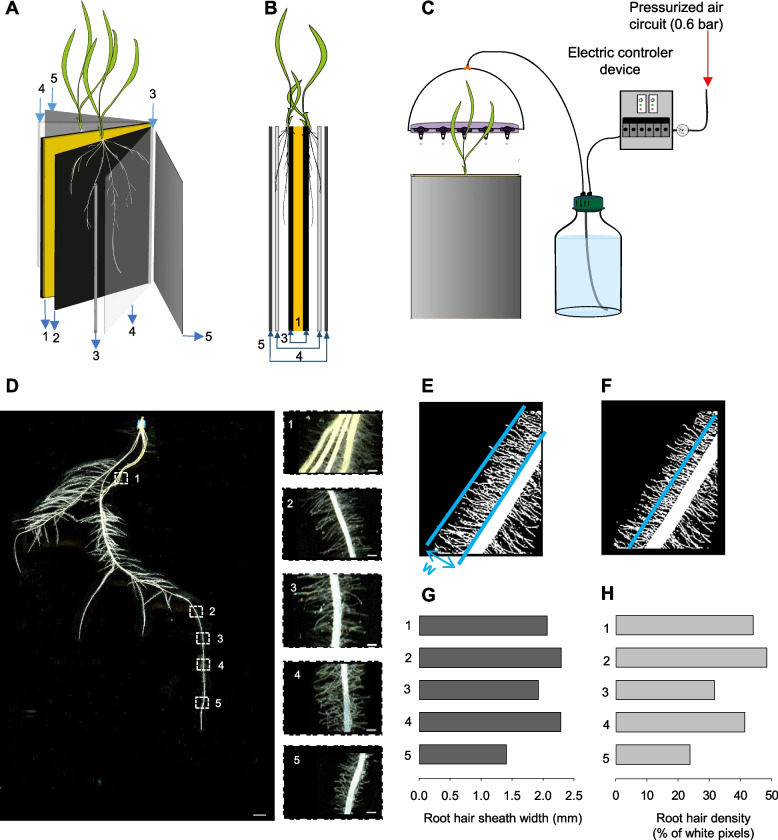
Integrative phenotyping of root hair development throughout the wheat root system in a rhizobox-type device. **A-C** The rhizobox-type device developed for integrative phenotyping of root hair development in whole root system. **A**, **B** Arrangement of the different rhizobox components: side view (**A**) and corresponding cross section (**B**). (1) Solid polyurethane foam panel. (2) Black polyester fabric bagging the polyurethane foam panel. (3) U-shape aluminum sliding bar. (4) Transparent glass plate. (5) Opaque polystyrene plate protecting roots from light. The rhizobox usually contains two plants grown simultaneously, one on each side of the rhizobox between the external face of the fabric and the glass plate. The aluminum sliding bars are used as spacers, setting the distance between the polyester fabric and the glass plate at 2 mm. The polyurethane foam panel acts as a nutrient solution reservoir, periodically replenished by the dedicated irrigation device and maintaining the fabric moistened. **C** The irrigation device ensuring controlled delivery of nutrient solution to the growing seedlings. The nutrient solution is contained in a 2- or 5-l bottle, which is fitted with a symmetrical plastic tube circuit, comprising 5 taps allowing the solution to drop onto the top of the polyurethane foam panel. Periodic pressurization of the bottle content with air (0.6 bar) ensures periodic flow of nutrient solution from the bottle to the taps. A controller (including timers and 3-way solenoid valves) is used to periodically pressurize/depressurize the bottle. In the present experiments, we set the duration of each watering episode to 3 s, every 10 min, delivering about 1.5 ml per watering episode and per rhizobox. **D-H** Representative image of a wheat root system grown in the rhizobox-type device and focus on root hairs at different regions along a seminal root. **D** Image of the entire root system of a Oued Zenati plant grown for 14 days in the rhizobox-type device. The plant was watered with 6 mM K^+^-containing nutrient solution. Five regions of the longest seminal root (numbered 1 to 5, chosen at varying distances from the tip, 18, 8, 7, 5.5 and 2.5 cm, respectively) are magnified in the right-side panels. **E** Operational estimation of average root hair length. The distance (named w) between a line drawn along the surface of the root segment and a second line which crosses the tips of the longest root hairs in the image is taken as an operational estimate of the average root hair sheath width in the corresponding root segment. **F** Operational estimation of average root hair density. The number of root hairs crossed by a line drawn parallel to the root surface at a distance of about w/2 (i.e. half of the root hair sheath width; see above) can be considered to be proportional to the number of white pixels crossed by the line (see “Image analysis” in the Material and methods for further details on its determination) since all root hairs appear to have the same diameter. **G-H** Root hair sheath width (G) and root hair pixel density (H) measured in the 5 regions magnified in panel D. For regions 2 to 4, the data provide the mean of the values obtained on both sides of the root segment

The rhizobox-type device described in Fig. 1A-C was developed in order to acquire images allowing to phenotype whole root systems of young plants with a high-enough resolution to include root hairs. A representative image of a durum wheat root system grown for 14 days in such a rhizobox-type device watered with a complete nutrient medium (modified Hoagland) is shown in Fig. 1D, together with enlargements of 5 regions of the longest seminal root, showing root hairs (right lateral panels).

From this type of images, operational analyses of root hairs were implemented, providing estimates of local root hair length and density (Fig. 1E and F). Such analyses were applied to approximately 4-mm-long root segments at the 5 locations along the seminal root shown in Fig. 1D. Long root hairs were present in each of the 5 analyzed regions (including old and young root regions). The width of the "root hair sheath" (operational root hair length estimate), however, substantially varied between regions (from ca. 1.4 mm to 2.3 mm; Fig. 1G), providing evidence that quite large heterogeneities in root hair length can occur over short distance along a root segment (15% and 38% difference between region 4 and the adjacent regions 3 and 5, respectively, distant by 1 or 2 cm). The operational estimates of root hair density (Fig. 1H) also provided evidence of heterogeneities in root hair density over short distances along this seminal root segment. Heterogeneities in local values of root hair length and density were observed in every root system image (see also the insets in Fig. 4).

### Effect of K^+^ shortage on plant K^+^ nutrition, root system development and shoot biomass in two wheat accessions grown in the rhizobox-type device

**Fig. 2:**
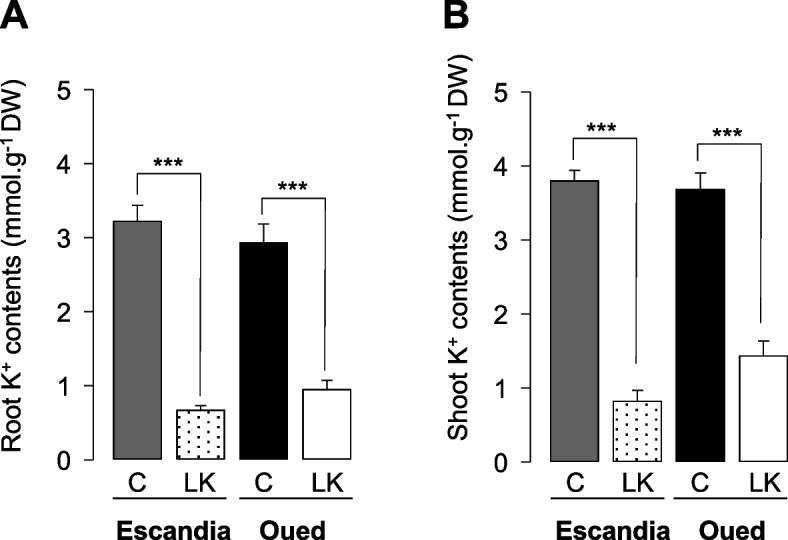
Effects of K^+^ shortage on K^+^ contents in Escandia and Oued Zenati wheat seedlings grown in rhizobox-type devices. Escandia and Oued Zenati wheat seedlings were grown in rhizoboxes for 14 days. The modified Hoagland nutrient solution contained either 6 mM (control (“C”)) or 60 µM K^+^ (low K^+^ (“LK”) condition). Root (**A**) and shoot (**B**) K.^+^ contents were measured by flame spectrophotometry. Means ± SE (*n* = 14 for Oued Zenati and 6 for Escandia). *** indicates that the difference between the values in control condition and the corresponding values in LK condition is statistically significant (Student *t* test; *p* < 0.005)

The rhizobox-type device was used to examine the consequences on root system development of K^+^ shortage. Two *Triticum turgidum* L. subspecies were grown in parallel, a durum wheat landrace, Oued Zenati, and an ancestral *dicoccum* genotype, Escandia. Plants were watered with either a control nutrient solution, containing 6 mM K^+^, or a low K^+^ solution containing 60 µM K^+^. The root and shoot K^+^ contents were assayed after 14 days of growth in the rhizobox-type devices (Fig. 2). The low K^+^ treatment resulted in strong decrease in K^+^ contents of both roots and shoots, when compared with the control treatment, in both Oued Zenati and Escandia wheat plants (Fig. 2). The root K^+^ contents were decreased by at least 3 times, from *ca.* 3 mmol.g^−1^ DW to 1 mmol.g^−1^ DW or less in the two wheat genotypes (Fig. 2A) or, when expressed on fresh weight basis, from *ca.* 200 µmol.g^−1^ FW to 40 µmol.g^−1^ FW or less (root DW to FW ratio close to 5%). The shoot K^+^ contents were reduced from *ca.* 3.7 mmol.g^−1^ DW (400 µmol.g^−1^ FW) to 1.5 mmol.g^−1^ DW (80 µmol.g^−1^ FW) or less than 1 mmol.g^−1^ DW (< 50 µmol.g^−1^ FW) in the two wheat genotypes (Fig. 2B; shoot DW to FW ratio close to 10%).

**Fig. 3:**
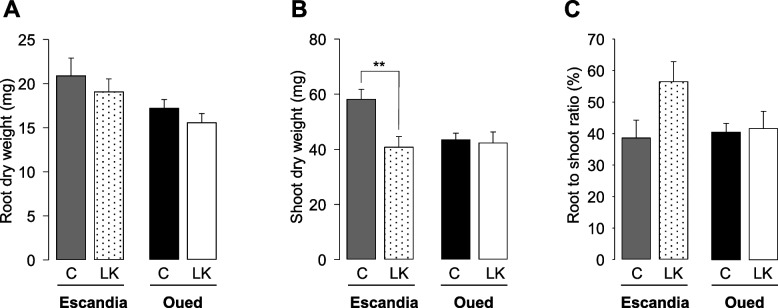
Effects of K^+^ shortage on root and shoot biomass in Escandia and Oued Zenati wheat seedlings. Escandia and Oued Zenati wheat seedlings were grown in rhizoboxes for 14 days in the same ionic conditions (control and low K^+^) as in Fig. 2. **A** Root, and **B** shoot biomass (dry weight: DW), and **C** root to shoot DW ratio (calculated from **A** and **B**). Means ± SE (*n* = 10 for control condition, and 14 for low K.^+^ condition, in Escandia; *n* = 14 for both conditions in Oued Zenati). ** denotes statistically significant differences (Student *t* test; *p* < 0.01)

The root biomass was not significantly affected by the K^+^ shortage treatment in either wheat genotype (Fig. 3A). The production of shoot biomass in Oued Zenati was also not affected by the K^+^ shortage treatment (Fig. 3B). In contrast, in Escandia, this treatment resulted in a significant reduction in shoot biomass, by *ca.* 25%, and thus in an increase in root to shoot biomass ratio (Fig. 3B and C).

Since it could be estimated that after 14 days of growth in the rhizoboxes, the plant K^+^ content originated essentially from K^+^ uptake from the watering solution even in low K^+^ conditions (the grain provided at most 10% of plant K^+^, knowing that the wheat grains contained ca. 240 µg K^+^; Figs. 2 and 3, and White et al. 2021), the efficiency of K^+^ uptake (KUpE: plant K^+^ per available K^+^ unit) was determined in Oued Zenati and Escandia plants subjected to low K^+^ treatment (Fig. S1A). No significant difference in KUpE was observed between the two genotypes (Fig. S1A). K^+^ use efficiency of both genotypes (KUE: biomass produced per available K^+^ unit) and the ability of both genotypes to utilize K^+^ for vegetative growth (KUtE: biomass produced per plant K^+^ unit) were also determined (Fig. S1B and C) in the two genotypes and did not appear significantly different either.

**Fig. 4:**
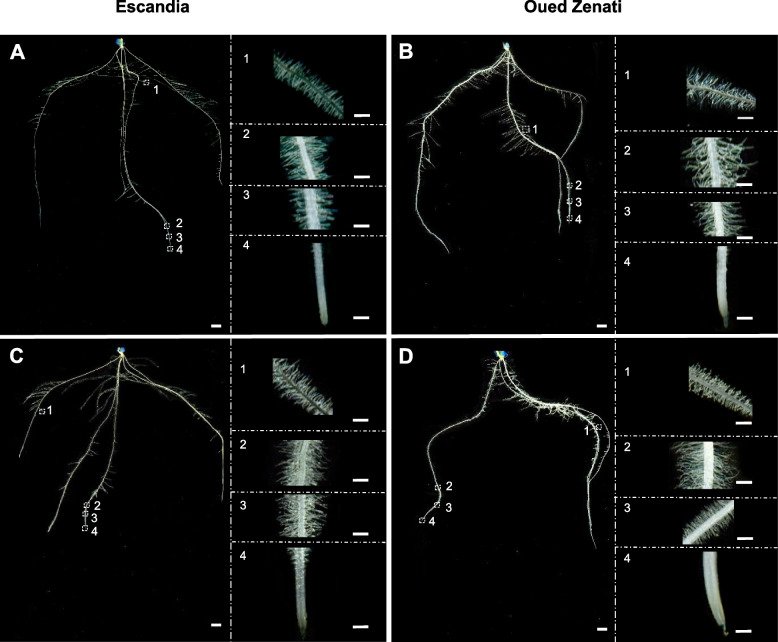
Representative images of root systems of Escandia and Oued Zenati wheat seedlings grown in the rhizobox-type device under control or K^+^ shortage conditions. Plants were grown for 14 days in rhizobox devices in control (**A**, **B**) or K^+^ shortage (**C**, **D**) conditions (see legends to Figs. 1 and 2). **A, C** Escandia; **B, D** Oued Zenati. Insets at right of panels show enlargements of regions from seminal and lateral roots. White bars: 1 cm for the whole root systems, and 1 mm for the enlargements

**Fig. 5:**
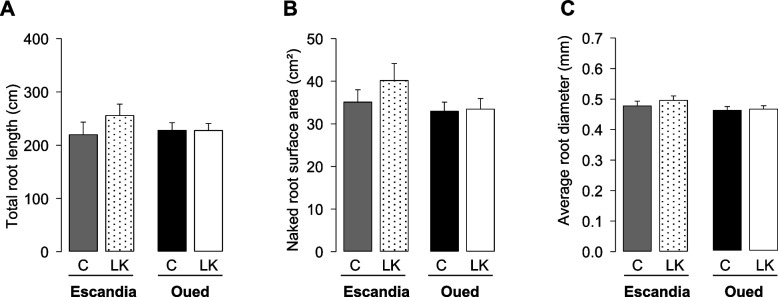
Effect of K^+^ shortage on total length of root system, on "naked" root surface area and on average root diameter in Escandia and Oued Zenati wheat seedlings. Plants were grown for 14 days in rhizoboxes in control or K^+^ shortage conditions (see the legend to Fig. 2). **A** Total root system (seminal + lateral root) length, obtained using WinRHIZO™ software. Root elements displaying a diameter equal to or larger than 0.2 mm were classified as roots, in contrast with root hairs that were assimilated to root elements with a diameter smaller than 0.2 mm. **B** “Naked” (when root hairs were not considered) root surface area, obtained using WinRHIZO™software. **C** Averaged root diameter over the entire root system (expressed in mm), calculated by dividing the “naked” root surface area (data in Fig. 5B) by the total root length (data in Fig. 5A) and by π (3.14). Means ± SE (*n* = 10 for control condition, and 14 for low K^+^ condition, in Escandia; *n* = 14 for both conditions in Oued Zenati). Absence of star above the bars indicates no statistically significant difference between the values in control condition and the corresponding values in K.^+^ shortage condition (Student *t* test; *p* > 0.05)

Representative images of Oued Zenati and Escandia root systems developed for 14 days in rhizobox-type devices supplied with control or low K^+^ solutions, are shown in Fig. 4. Total length and surface area of the “naked” root system (when root hairs were not considered) were determined over the entire root system (Fig. 5). Total length (Fig. 5A) and surface area (Fig. 5B) of the “naked” root system were not significantly affected by the K^+^ shortage treatment in Oued Zenati and in Escandia. The average root diameter over the whole root system was also unaffected by the K^+^ shortage treatment in both wheat accessions (Fig. 5C).

### Effect of K^+^ shortage on local root hair production in seminal and lateral roots of the two wheat accessions

**Fig. 6:**
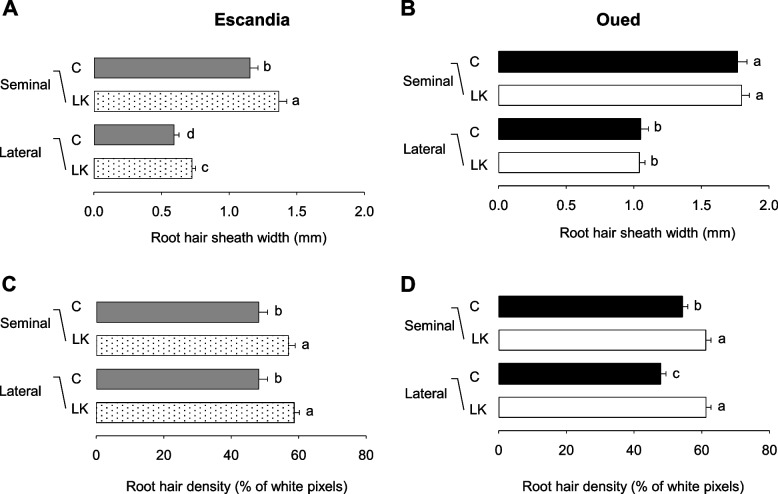
Differences in local root hair elongation and density values in wheat plants grown in control or K^+^ shortage conditions. Escandia and Oued Zenati wheat plants were grown for 14 days in rhizoboxes in control and low K^+^ conditions (see the legend to Fig. 2). Operational estimates of root hair elongation ("root hair sheath width") and of root hair density ("root hair pixel density" along a line parallel to the root surface) were obtained from the same plants as in Fig. 4 and 5 according to the procedures described in Fig. 1D and F, respectively. Measurements were performed at 6 cm from the root tip for seminal roots, and at the middle of lateral roots, over 4 mm root segments in both cases. Segments from 4 seminal roots and 4 lateral roots from different seminal roots were analyzed per plant. **A, B** Root hair sheath width in Escandia (**A**) and Oued Zenati (**B**) seminal and lateral roots of plants grown under control or low K^+^ conditions. **C, D** Root hair (white pixels) density in Escandia (**C**) and Oued Zenati (**D**) seminal and lateral roots of plants grown under control or low K^+^ conditions. Means ± SE (*n* = 40 from 10 plants for control condition and 56 from 14 plants for low K^+^ condition in Escandia; *n* = 56 from 14 plants for both conditions in Oued Zenati). Different letters denote statistically significant differences (One way anova test, *p* < 0.05) between the values in control condition and the corresponding values in K^+^ shortage condition, and between values in seminal and lateral roots

Additional analyses were carried out to measure root hair length and density in different root types of the root systems. Both seminal and lateral roots were analyzed (see examples of root hair images from both types of roots in Fig. 4). The measurements were carried out using the procedures described in Fig. 1E and F. To allow comparison between the two wheat genotypes, since we observed that root hair length and density showed significant heterogeneities with position along the same root, especially in the youngest regions close to the root tip (Fig. 1G and H, Fig. 4), the latter measurements were performed on 4-mm-long regions at fixed positions, selected *ca.* 6 cm from the root tip in seminal roots and in the middle of the root in lateral roots. The resulting values of root hair length were higher in seminal than in lateral roots in both wheat genotypes (Fig. 6). In contrast, root hair density appeared to be weakly dependent on root type in these measurements (Fig. 6).

In Escandia plants grown under K^+^ shortage conditions compared with the plants grown under control conditions, root hair length values were higher, by ca. 20% in both seminal and lateral roots (Fig. 6A). Escandia root hair density values were also greater in plants grown under K^+^ shortage conditions, also by about 20%, in both root types, compared with plants grown under control conditions (Fig. 6A). In Oued Zenati, root hair density was found similarly to be increased by the K^+^ shortage treatment, but root hair length was not affected (Fig. 6B).

### Root hair contribution to entire root system area and response to K^+^ shortage

We then sought to obtain overall estimates of root hair area in the root systems. Such data could be obtained directly using WinRHIZO™ after image segmentation thanks to the high resolution of the root system images (Fig. 4), with root hairs then considered in WinRHIZO™ as the finest class of roots (with diameters less than 0.2 mm; see Materials and methods).

**Fig. 7:**
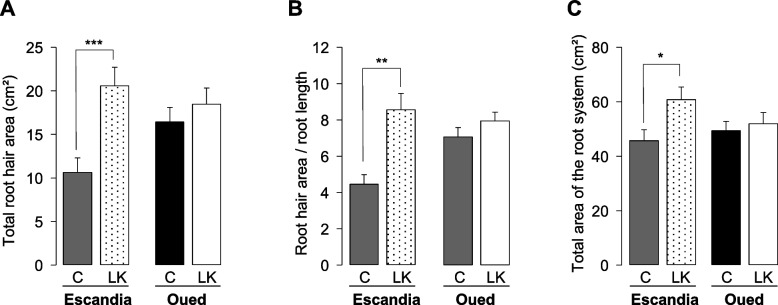
Effect of K^+^ shortage on root system and root hair surface areas in Escandia and Oued Zenati wheat seedlings. Plants were grown for 14 days in rhizoboxes in control or K^+^ shortage conditions (see legend to Fig. 2). Plants were the same as in Figs. 5 and 6. **A** Total root hair surface area. **B** Ratio of total root hair surface area (cm^2^) to total root (seminal + lateral) length (cm), as an average root hair surface over the root system. Total root (seminal + lateral) length data were from Fig. 5A). **C** Entire root system surface area (roots + root hairs). Areas were measured using WinRHIZO™, the diameter classification parameters being set so that root hairs were assimilated to root elements with a diameter smaller than 0.2 mm. Root elements displaying a diameter equal to or larger than 0.2 mm were classified as roots. Means ± SE (*n* = 10 for control condition, and 14 for low K^+^ condition, in Escandia; *n* = 14 for both conditions in Oued Zenati). *, ** and *** denote statistically significant differences (Student *t* test; *p* < 0.05, 0.01 or 0.005, respectively) between the values in control condition and the corresponding values in K^+^ shortage condition

The K^+^ shortage treatment resulted in a large increase in total root hair area in the Escandia root system (Fig. 7A), consistent with the locally evidenced increases in both root hair length and density (Fig. 6). In contrast, although local measurements in Oued Zenati indicated increase in one of the root hair parameters under K^+^ shortage (density; Fig. 6), only a non-significant slight increase in root hair area was found over the entire root system of this accession (Fig. 7A).

Total root hair area values were then divided by total root system lengths to determine the extent to which changes in total root hair areas under K^+^ shortage conditions were due to changes in the ratio of root hair to “naked root” production rather than in the development of the entire root system. The ratio of root hair area to root length showed a similar variation to that of root hair area in both wheat varieties under K^+^ deficiency (Fig. 7B). Thus, while no significant change in the ratio of root hair area to root length was observed in Oued Zenati, an increase greater than 100% was observed in Escandia (Fig. 7B). This indicates that the developmental program of the root system in Escandia was strongly modified by the K^+^ shortage treatment, with a strong increase in the root hair to “naked root” ratio.

Finally, the sensitivity of root hair production to K^+^-shortage in Escandia was assessed at the whole root system level by comparing the surface of the whole root system (including root hairs) under control and low K^+^ growth conditions. K^+^ shortage resulted in Escandia in a substantial increase in the surface area of the entire root system (by ca. 35%; Fig. 7C). Similar analyses in Oued Zenati indicated, in agreement with the separate analyses of the root hairs and "naked" root system (Figs. 5B and 6B), that the total surface area of the root system was not significantly affected by the K^+^ shortage treatment in this wheat accession (Fig. 7C).

## Discussion

### Integrative analysis of the whole root system including root hairs

For K (but also for P and N), the changes in root system and root hair development in response to nutrient shortage have almost always been separately investigated, without integrative analyses (Bates and Lynch 1996; Wang et al. 2016; Klinsawang et al. 2018; De Pessemier et al. 2022). Thus, while root hairs have essential functions in nutrient uptake, and particularly in the case of K^+^ (Tanaka et al. 2014; Bienert et al. 2021), no assessment of root hair contribution to the total root system surface area and how this parameter varies with external nutrient availability was generally attempted. Furthermore, root hair analyses so far concerned selected region(s) of the root system relatively restricted in length but implicitly taken as representative of the whole root system (Lamont 1983; Høgh‐Jensen and Pedersen 2003; Wang et al. 2016; Vincent et al. 2017).

Here, we have investigated the developmental responses of the root system and root hairs to K^+^ shortage in wheat, in two strongly different genetic backgrounds, a wheat ancestor and a durum wheat landrace. We first assessed the variability of root hair length and density in the root systems, along the roots and between root types (Fig. 1D-H, Fig. 5), which revealed very significant variability, especially in the length of root hairs between seminal and lateral roots (Fig. 6). Next, our goal was to perform integrated analyses of root hair responses to K^+^ deficiency treatment within the whole root system (Fig. 7A), and then to perform further integration to access altogether the “naked roots” and root hairs in terms of uptake surface area, in our analysis of root system responses to K^+^ deficiency treatment (Fig. 7C). This required the development of a dedicated rhizobox-type device and an imaging procedure.

The rhizobox-type device (Fig. 1A-C) actually allowed to obtain well resolved images of root hairs, in terms of length and density, on entire root systems (Fig. 1D-F and Fig. 4). The suitability of this experimental device for such investigation essentially results from the fact that root growth occurs at the surface of a black (increasing the contrast between the roots and the background) polyester fabric (non-putrescible and kept moistened by a polyurethane foam panel periodically watered) at a constant and adequately chosen distance (2 mm in the present experiments with wheat) from a glass plate. This allowed to directly get high resolution scans, through the glass plate, of root systems and their root hairs. The quality of the segmented images, which seems without equivalent in the literature to our knowledge, allowed to carry out integrative analyses of the plant responses to K^+^ shortage, in terms of both root system development and root hair production, using the classical softwares ImageJ and WinRHIZO™ (Thornburg et al. 2020; Strock et al. 2018; Saengwilai et al. 2014; Vandamme et al. 2013).

### Relevance of the root hair trait parameters in the analysis of plant response to K^+^ shortage

In most soil, K^+^ is poorly mobile due to strong interactions (adsorption/desorption) with soil constituents, notably clay minerals and organic matter (Sparks and Huang 1985; Sparks 1987; Dobermann et al. 1998; Zörb et al. 2014). It is widely recognized that important adaptive responses to low K^+^ (a common situation in the absence of agricultural inputs) lie in functional adjustments of the root system to increase its uptake capacity, which can be achieved, in particular, by increasing the surface area of the root system and/or by inducing or enhancing the activity of the outer root layer K^+^ transport systems. The combination of strategies adopted varies among species and accessions. For example, some tomato varieties found to be tolerant to K^+^ deficiency compared to susceptible varieties were observed to increase their root surface uptake capacity, while others enhanced the growth of their root system (Chen and Gabelman 2000).

Root ideotypes likely to enhance, under scarcity conditions, the uptake of nutrients such as K^+^ that crops require in large quantities, have been proposed. Such root traits include greater production of lateral roots and root hairs to increase the surface area for K^+^ uptake by roots and to reduce the distance over which K^+^ diffusion and water mass flow to peripheral root cells take place (White et al. 2013). Root hair parameters (length and density) are thus commonly mentioned along with the length of the roots, as likely major determinants of the acquisition of poorly mobile nutrients such as K^+^ (Mengel and Steffens 1985; Sustr et al. 2019; Bienert et al. 2021). Molecular information on cell-specific expression of K^+^ transport systems in the outer layers of roots, with large expression/activity in root hair cells of both low-affinity Shaker-type K^+^ uptake channels and K^+^ shortage-induced high-affinity HAK K^+^ transporters (Véry et al. 2014; Damiani et al. 2016), actually pointed to the root hair as a likely major contributor to root K^+^ uptake, in agreement with analyses of developmental mutants affected in root hair elongation in Arabidopsis (Rigas et al. 2001; Tanaka et al. 2014; e.g., 40% loss of K^+^ uptake in the Arabidopsis *trh1* mutant with very short root hairs). It appears therefore highly relevant when trying to analyse the absorption capacity of a root system to integrate the total surface area of the root hairs in the root system with that of the "naked root bodies". Priority was thus given in the present study to the analysis of the effects of K^+^ shortage on total root surface area (including root hairs) and contribution of root hair area to the area of the whole root system (Figs. 5B, Fig. 7A and C).

The heterogeneities in root hair length and density that were observed at the entire root system level, for instance along a long seminal root (Fig. 1G and H, Fig. 4) or between lateral and seminal roots (Figs. 4 and 6) were present in both root systems developed in the high or low K^+^ condition (Fig. 1G and H; Fig. 4) and are therefore not directly related to the plant response to K^+^ availability. It should be noted, for instance, that in Arabidopsis grown in Petri dishes on agar plates devoid of carbon source, root hair elongation has been reported to be much reduced during the dark period, when compared with the light period (Poitout et al. 2017). Thus, global parameters such as the whole root hair area (Fig. 7A) or the whole root system area (roots + root hairs; Fig. 7C), avoid any bias in the selection of local zones to measure root hair length and density, and thereby provide valuable information on the integrated strategy of the plant to develop the whole area of its root system surface.

Root parameters describing root hair development on the root surface, such as total root hair area or local root hair density for example (Fig. 1F, Fig. 6C and D, Fig. 7A), should be considered as providing underestimated values of the corresponding trait because they are obtained from the 2-D projection (image of the root system) of a 3-D structure, the area of the projected root therefore masking partially that of root hairs. This bias is dependent on the diameter of the root, when compared to root hair length, the greater the diameter the larger the underestimation. K^+^ shortage did not affect the “mean” root diameter (averaged over the whole root system) in Escandia and in Oued Zenati (Fig. 5C). Furthermore, this parameter was very similar in Escandia and Oued Zenati (Fig. 5C). Thus, the changes in root hair area due to K^+^ shortage, as well as the differences between the two wheat accessions in their responses to this stress, essentially reflected changes in root hair production at the root surface.

In our wheat genotypes, the total root hair area (Fig. 7A) corresponded to 30 to 54% of the area of the "naked” root systems (Fig. 5B) under the different nutrient conditions and genotypes, and thus accounted for a very significant portion of the total root system area (22 to 40%; Fig. 7A and C), which is a very relevant parameter to include in the analysis of the development and function of the root system when considering nutrient uptake. In Oued Zenati, the contribution of the root hair surface area to the whole root system surface area did not significantly vary with the K^+^ treatment (33% in high K^+^ and 35% in low K^+^; Fig. 7A and C). In Escandia, in contrast, K^+^ shortage strongly increased the relative root hair area in the root system (which increased from 22% in high K^+^ to 40% in low K^+^; Fig. 7A and C). Thus, this indicates that strong developmental changes increasing the root hair to naked root surface area ratio can be induced in some wheat genotypes in response to K^+^ shortage stress. Furthermore, the fact that high variability between the two genotypes was observed in terms of reprogramming of root hair production upon low K^+^ treatment highlights the relevance of such integrative root hair surface analyses at the scale of the whole root system for the analysis of plant adaptation strategies to K^+^ shortage stress, in wheat and likely also in other species.

### Differences in responses to K^+^ shortage between Escandia and Oued Zenati wheat accessions

Low K^+^ treatments, compared to control conditions that can be considered as luxury feeding allowing an "insurance strategy", result in a sharp decrease in plant tissue K^+^ content and growth (biomass production). A possible critical concentration of K^+^ in plant tissues, between 0.5 and 2% of dry matter, has been proposed (Leigh and Wyn Jones 1984), but is recognized as difficult to assess since it obviously depends on the growth stage and the crop species (Zörb et al. 2014). Here, the 14-day low-K^+^ treatment (11 days with 60 µM K^+^ irrigation input after 3 days with deionized water input) in the rhizobox devices led to reduced K^+^ contents of root and shoot tissues in both genotypes down to levels consistent with those reported in wheat (*T. aestivum*) seedlings grown in hydroponics (Ruan et al. 2015), and slightly lower in Escandia than in Oued Zenati: 3% and 4% of root dry matter in Escandia and Oued Zenati, respectively, and 3.3 vs. 5% of shoot dry matter (Fig. 2). Since the K^+^ contents in well-fed plants were not significantly different between the two wheat varieties (Fig. 2), Escandia and Oued Zenati differed in terms of K^+^ homeostasis upon external K^+^ limitation. A stronger response to the K^+^ shortage treatment was observed in Escandia in terms of biomass allocation in favor to roots (Fig. 3C), a trait thought to improve K^+^ acquisition under this nutrient shortage condition (White et al. 2013; Sustr et al. 2019).

The global analyses of the root system development that were performed here led to the conclusion that the “naked” root system did not significantly change its overall absorbing area under K^+^ shortage in both wheat genotypes (Fig. 5). However, the K^+^ shortage induced a sharp increase in the root hair production of Escandia that very significantly increased the uptake area of the entire root system of this accession (Fig. 7), constituting another expected trait of adaptation to low K^+^ levels in this genotype. Oued Zenati, in contrast did not manifest overall developmental changes in response to the K^+^ shortage treatment (Figs. 3, 5, and 7). This indicates that the response to low K^+^ (sensing, adaptation ability/strategy) is different in the two varieties. It should be noted in particular that, although Oued Zenati appeared to be unresponsive to the K^+^ treatment, this variety showed growth parameters (root/shoot biomass) and root hair surface values under control conditions close to those observed in Escandia under low K^+^ conditions (Figs. 2 and 7). Thus, Oued Zenati seems to express independently of the availability of K^+^ in the external medium, some favorable traits to cope with K^+^ shortage. Finally, despite their very different response to changes in the external concentration of K^+^, both accessions showed under low K^+^ conditions very similar root hair areas (Fig. 7A, and B) and non-significantly different K^+^ uptake efficiency (Fig. S1A), indicating that, at least at this stage of their development, the two accessions could cope with low K^+^ availability with similar efficiencies, independently of their behavior under high K^+^ condition and their adaptation strategy to changes in K^+^ availability.

Very little information was yet available on the response of wheat roots to K^+^ availability and its variability among accessions. Variations in root and shoot biomass induced by K^+^ deficiency has been reported in hexaploid wheat varieties (Ruan et al. 2015), but no detailed analysis of root system development in response to external K^+^ availability was so far described to our knowledge. The integrated root hair analysis that we performed in this study, which provides quantitative (less subjective) estimates of the total root hair area in a whole root system, compared to analyses based solely on spots, appears to be particularly well suited for comparisons between conditions or genotypes. Since the variations in total root hair area appear to be substantial relative to those in "naked" root area, the integration of root hair area with "naked" root area thus provides a more relevant description of the total uptake area of root systems and its responses to nutrient availability. Such integrated analyses, whose contribution to a better description of root system responses to K^+^ stress has been highlighted here, are likely to provide valuable information on root adaptation to different nutrient stresses.

## Materials and methods

### Plant material and growth conditions

The effects of K^+^ deficiency on root system development were investigated in two wheat accessions, Oued Zenati (*Triticum turgidum *ssp*. durum*), which is a landrace, and Escandia (*Triticum turgidum *L*. *ssp*. dicoccum*), an ancestral genotype.

Seeds of similar size were selected and surface sterilized by immersion in 4% calcium hypochlorite solution for 20 min under vacuum (in order to remove the air bubbles possibly trapped in seed groove and impeding sterilization of the groove surface). The seeds were then rinsed 5 times in sterile distilled water and then soaked in sterile warm water (about 40 °C, and 20 ml for about 10 seeds) in a plastic pot, which was transferred to 26 °C in the dark for about 16 h. The latter treatment was found to stimulate seed germination.

Germinated seeds (seeds showing primary root emergence) were transferred to sterile moistened filter paper in a Petri dish and coated with a wax containing 0.1% oxyquinoline fungicide (Staehler PP 140, Chauvin-Agro) to prevent fungal contamination during the growth period. The plants were then grown in rhizobox-type devices (see below) developed in order to allow integrative phenotyping of root and root hair traits at the scale of the entire root system. Coated seeds were placed in the rhizobox devices with the emerged primary root facing downward. The rhizobox-type devices were randomly arranged in a growth chamber (16 h of photoperiod,150 μE of light intensity, day/night temperature 22/20 ˚C, 70% air humidity). Images of root systems were acquired after 14 days of growth.

### The rhizobox-type device

The rhizobox-type device allowing to obtain high-resolution images of root systems is described in Fig. 1A-C (see also the end of the first part of the Discussion section). A polyurethane foam panel (width/height/thickness in mm: 260/320/15, Smithers-Oasis France Sarl, Saint-Martin-Lalande; acting as nutrient solution reservoir: see below) is bagged in a black, hydrophilic, non-putrescible, water- and nutrient-permeable polyester fabric (mesh size: 18 μm, solution holding capacity: 2.5 µl/cm^2^; Saaticare Hyphyl®, Appiano Gentile, Italy). The polyurethane foam panel inside the rhizobox moistens the polyester fabric on which the roots develop (see below for watering device). The foam panel bagged in the fabric is placed between two transparent glass plates. Laterally placed aluminum spacers (2 mm thick and 20 mm wide) ensure a constant spacing between the glass plate and the black fabric. Each glass plate is covered with a polystyrene insulation panel that protects the growing root system from light (Fig. 1A and B). The roots grow between the polyester fabric and the glass plate. Two plants are grown simultaneously in one rhizobox, one plant on each side of the rhizobox. Images of the root system are acquired through the transparent glass plate. Nutrient solution is provided by an automatic device (Fig. 1C), with the solution dropped on top of the polyurethane foam panel (1.5 ml every 10 min), maintaining constant the water content of the panel.

The germinated seeds transferred to the rhizoboxes were first watered with deionized water for 3 days, and then the seedlings were watered for 11 days with either control (6 mM K^+^-containing) or low K^+^ (60 µM K^+^-containing) modified Hoagland solution (Shavrukov et al. 2012). The control solution comprised 5 mM KNO_3_, 1 mM KH_2_PO_4_, 1 mM NH_4_NO_3_, 2 mM MgSO_4_, 1 mM NaCl, 0.1 mM NaFe(III)EDTA, 12.5 µM H_3_BO_3_, 2 µM MnCl_2_, 3 µM ZnSO_4_, 0.5 µM CuSO_4_, 0.1 µM Na_2_MoO_4_, and 0.1 µM NiSO_4_. The low K^+^ solution contained 60 µM KH_2_PO_4_ as sole source of K^+^, MgSO_4_ and micro-nutrients introduced at the same concentration as in the control solution, and 5 mM NaNO_3_ and 1 mM NaH_2_PO_4_, these two sodium salts replacing/compensating the absence/decrease in concentration of the corresponding potassium salts in the control solution (5 mM added Na^+^ to a basis of 1 mM present in the control solution being unlikely to have a significant physiological/developmental impact; Thorne and Maathuis 2022).

### Image acquisition and segmentation

Images of 14-day old root systems were acquired though the transparent glass plates with a high-resolution flatbed scanner (Epson Perfection V850 pro; resolution 24 bits/1200 dpi). The obtained root images were processed using the Ilastik software (Berg et al. 2019) in order to perform root segmentation from the background.

The segmentation was performed using the predefined “pixel classification workflow” of the Ilastic software. We trained the program to distinguish the background (pattern of the fabric) from the roots and root hairs using manual annotation/labeling. The selected parameters in the Ilastik software were: "Gaussian Filter, Color/Intensity, Edge, and Texture", with sigma value less than 0.7 to extract fine details like root hairs. This process resulted in eight-bit segmented images, which could be directly analyzed by bio-image analysis softwares.

### Image analysis

Segmented root images were analyzed using WinRHIZO™ (V.2009 Pro, Regent Instruments, Montreal, QC, Canada) and ImageJ (version 1.53) (Abràmoff et al. 2005). Morphometric root traits such as total root length, root surface area and root hair surface area were directly obtained through WinRHIZO™ analysis (Bauhus and Messier 1999). Two classes of diameters were defined within WinRHIZO™ (user definition): diameter smaller than 0.20 mm, or larger than 0.2 mm. Root elements identified as belonging to the thinner diameter class (0 to 0.20 mm) were considered as corresponding to root hairs. The 0.2 mm threshold was chosen following tests taking into account that the projected area of several root hairs can overlap in scans of root systems. It was the largest threshold that allowed to efficiently exclude roots from the root hair class. The average root diameter over the whole root system was obtained by dividing the “naked” root system surface area by the length of the corresponding root system and by π (3.14) in agreement with the WinRHIZO procedure (given that the root total surface area obtained using WinRHIZO = ∑[local estimates of root diameter x length of the corresponding root element x π]). At last, operational estimates of root hair extension and density were directly obtained using ImageJ according to the procedure described in Fig. 1E and F, respectively. For the operational estimate of root hair density, the density D% of the white pixels intercepted by the line drawn at the distance of about half of the root hair sheath width was determined. D% is the number of white pixels reported (in %) to the total number of pixels, white or black, intercepted by this line. D% was obtained directly from the "mean gray value", m_gray_, obtained using ImageJ for the pixels intercepted by the line. In the 8-bit coded image (white and black pixels color code: 255 and 0, respectively), D% = 100 x m_grey_/255.

### Biomass and K^+^ content analyses

Wheat plants were sampled for biomass measurements and K^+^ content analyses immediately after acquisition of the root system images at the end of the 14-day growth period in rhizoboxes. Shoot and root fresh weights (FW) were measured. Dry weights were determined after 72 h at 60 °C. Shoot and root potassium contents were assayed by flame spectrophotometry (SpectrAA 150220FS, Varian) after ion extraction from dry tissue samples with 0.1 N HCl (Sentenac and Grignon 1981).

### Statistical analyses

Statistical analyses were carried out using SigmaPlot Version 11 (Systat Software, Inc). Data are presented as means and standard errors of the distributions. Student’s t-test or analysis of variance (ANOVA) were performed to determine the significance of differences between data sets. All tests were two-tailed, performed at the significance level α = 0.05. For all analyses, *p* < 0.05 was considered statistically significant (*p < 0.05; **p < 0.01, ***p < 0.005).

## Supplementary Information

**Additional file 1: Fig. S1.** Efficiency of K^+^ uptake (KUpE, plant K per available K^+^ unit; A), of K^+^ use (KUE, plant biomass per available K^+^ unit; B) and of K^+^ utilization (KUtE, plant biomass per plant K^+^ unit; C) under K^+^ shortage conditions in Escandia and Oued Zenati wheat plants grown in rhizobox-type devices. Wheat seedlings were grown for 14 days in rhizoboxes watered with a modified Hoagland nutrient solution containing 60 µM K^+^. Whole plant K^+^ content (measured by flame spectrophotometry) and biomass used for the calculation of KUpE, KUE, and KUtE (White et al. 2021), were determined from the root and shoot data presented in Figs. 2 and 3, respectively. Means ± SE (*n* = 6 for Escandia and 14 for Oued Zenati). Absence of star above the bars indicates that the difference between the values in Escandia and the corresponding values in Oued Zenati is not statistically significant (Student *t* test; *p* > 0.05).

## Data Availability

The data and material that support the findings of this study are available from the corresponding author upon reasonable request.

## Abbreviations

2-D/3-D: 2-Dimensions/3-dimensions
ca.: Circa
DW: Dry weight
FW: Fresh weight
p: Probability
vs.: Versus
