# Supplementary figures and images for: Plasticity of wheat seedling responses to K+ deficiency highlighted by integrated phenotyping of roots and root hairs over the whole root system
Source: Stress Biol. 2023 Apr 6;3(1):5. doi: 10.1007/s44154-023-00083-4 (PMC10441938; doi:10.1007/s44154-023-00083-4)

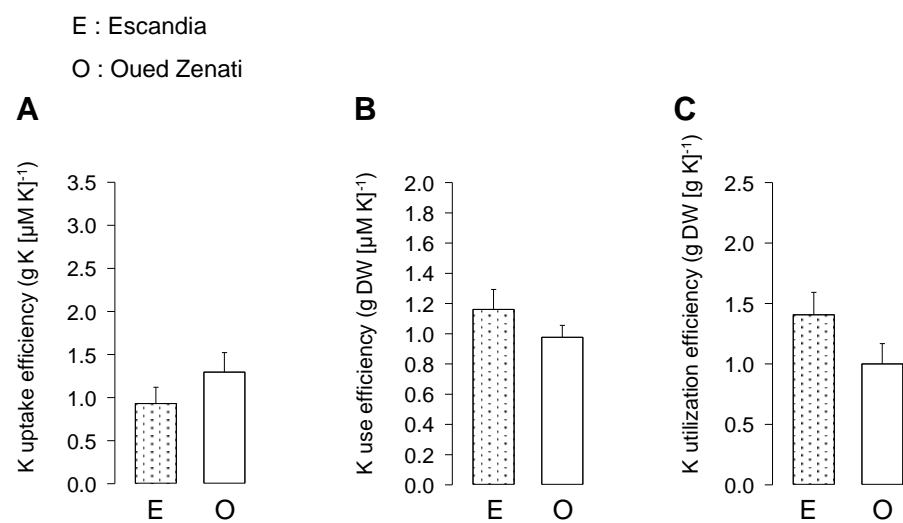

Fig. S1

Supplement: Supplementary file 1 — Additional file 1: Fig. S1. Efficiency of K+ uptake (KUpE, plant K per available K+ unit; A), of K+ use (KUE, plant biomass per available K+ unit; B) and of K+ utilization (KUtE, plant biomass per plant K+ unit; C) under K+ shortage conditions in Escandia and Oued Zenati wheat plants grown in rhizobox-type devices. Wheat seedlings were grown for 14 days in rhizoboxes watered with a modified Hoagland nutrient solution containing 60 µM K+. Whole plant K+ content (measured by flame spectrophotometry) and biomass used for the calculation of KUpE, KUE, and KUtE (White et al. 2021), were determined from the root and shoot data presented in Figs. 2 and 3, respectively. Means ± SE (n = 6 for Escandia and 14 for Oued Zenati). Absence of star above the bars indicates that the difference between the values in Escandia and the corresponding values in Oued Zenati is not statistically significant (Student t test; p > 0.05). [file 44154_2023_83_MOESM1_ESM.pdf]
